# The N-Terminus of GalE Induces tmRNA Activity in *Escherichia coli*

**DOI:** 10.1371/journal.pone.0015207

**Published:** 2010-12-07

**Authors:** Zachary C. Ruhe, Christopher S. Hayes

**Affiliations:** 1 Department of Molecular, Cellular and Developmental Biology, University of California Santa Barbara, Santa Barbara, California, United States of America; 2 Biomolecular Science and Engineering Program, University of California Santa Barbara, Santa Barbara, California, United States of America; University of Edinburgh, United Kingdom

## Abstract

**Background:**

The tmRNA quality control system recognizes stalled translation complexes and facilitates ribosome recycling in a process termed ‘ribosome rescue’. During ribosome rescue, nascent chains are tagged with the tmRNA-encoded SsrA peptide, which targets tagged proteins for degradation. In *Escherichia coli*, tmRNA rescues ribosomes arrested on truncated messages, as well as ribosomes that are paused during elongation and termination.

**Methodology/Principal Findings:**

Here, we describe a new translational pausing determinant that leads to SsrA peptide tagging of the *E. coli* GalE protein (UDP-galactose 4-epimerase). GalE chains are tagged at more than 150 sites, primarily within distinct clusters throughout the C-terminal domain. These tagging sites do not correspond to rare codon clusters and synonymous recoding of the *galE* gene had little effect on tagging. Moreover, tagging was largely unaffected by perturbations that either stabilize or destabilize the *galE* transcript. Examination of GalE-thioredoxin (TrxA) fusion proteins showed that the GalE C-terminal domain is no longer tagged when fused to an N-terminal TrxA domain. Conversely, the N-terminus of GalE induced tagging within the fused C-terminal TrxA domain.

**Conclusions/Significance:**

These findings suggest that translation of the GalE N-terminus induces subsequent tagging of the C-terminal domain. We propose that co-translational maturation of the GalE N-terminal domain influences ribosome pausing and subsequent tmRNA activity.

## Introduction

Bacteria possess several molecular quality control systems to ensure the fidelity of protein synthesis. The tmRNA•SmpB quality control system functions in all eubacteria to recycle stalled translational complexes in a reaction termed ‘ribosome rescue’. tmRNA (transfer-messenger RNA) is a bi-functional RNA that acts first as a transfer RNA to bind the ribosomal A site, and then as a messenger RNA to add the SsrA peptide tag to the C-terminus of the nascent chain [1]. SmpB is a tmRNA-binding protein required for both ribosome binding and translation of the SsrA peptide [2], [3]. The tmRNA•SmpB system serves at least two other quality control functions in addition to ribosome rescue. First, tmRNA•SmpB activity releases truncated or damaged messages, thereby facilitating their rapid turnover [4], [5]. Second, the SsrA peptide is a degradation signal that targets tagged polypeptides to a number of proteases [1], [6], [7], [8]. These activities ensure that defective mRNAs and proteins are identified and destroyed, thereby reducing the burden of non-productive protein synthesis.

tmRNA•SmpB activity was first demonstrated with ribosomes stalled at the 3′-ends of truncated, or ‘non-stop’ messages, which lack in-frame stop codons [1]. Non-stop transcripts can be generated by ribonuclease activity [5], [9], [10], [11] and premature transcription termination [12], [13]. In addition, there is at least one instance of a naturally encoded non-stop mRNA [14]. In *Caulobacter crescentus*, tmRNA•SmpB activity is induced by a 16-nucleotide sequence element found in several genes [15]. Remarkably, insertion of this element into either the template or non-template DNA strand results in tagging of encoded proteins. In *Escherichia coli*, tmRNA•SmpB acts on ribosomes paused at clusters of non-preferred, or “rare”, codons [16], [17], [18]. Rare codons are typically decoded by low abundance tRNAs, and therefore ribosomes are thought to pause during the translation of these codons. Additionally, ribosome pausing at stop codons during inefficient translation termination leads to tmRNA•SmpB activity in *E. coli* [19], [20], [21]. tmRNA•SmpB does not act at ribosomes stalled on full-length mRNA [22], so transcripts must first be processed into truncated forms before ribosome rescue can occur at rare codons and stop codons. Together, these observations support a model in which tmRNA•SmpB monitors translation and responds specifically to stalled ribosomes.

In this communication, we propose a new ribosome pausing mechanism that induces SsrA tagging activity. The *E. coli* GalE protein (UDP-galactose 4-epimerase) is tagged at more than 150 distinct sites, primarily within the C-terminal domain. This tagging was largely unaffected by the deletion, and overproduction, of ribonucleases involved in mRNA degradation. Moreover, synonymous recoding of the last 170 codons within *galE* had little effect on tagging. These data suggest that codon usage, mRNA structure, and RNase activity are not primary determinants of GalE tagging. Examination of fusions between GalE domains and thioredoxin (TrxA) showed that the N-terminus of GalE induces SsrA tagging within the fused C-terminal TrxA domain. In contrast, the GalE C-terminal domain was not tagged when fused to TrxA. We propose a model in which co-translational maturation of the N-terminal domain influences synthesis of the GalE C-terminal domain. Such a mechanism would target defective proteins for degradation after release from the ribosome.

## Results

### GalE is tagged at several sites

**Figure 1:**
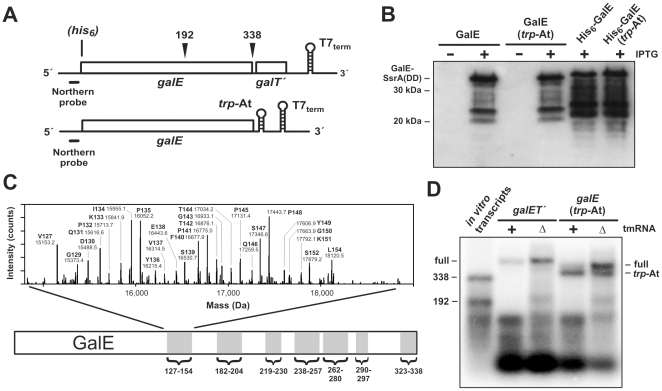
GalE is tagged with the SsrA peptide at several sites. **A**) Schematic representation of the *galE* transcripts used in this study. GalE was produced from constructs with and without downstream *galT́* coding sequence. His_6_-GalE variants were also expressed from related constructs encoding an N-terminal hexa-histidine (*his_6_*) epitope tag. The intrinsic transcription terminator from the *E. coli trp* attenuator (*trp-*At) was introduced downstream of *galE* as described in the Methods. Northern blot probe binding sites and the positions of codon-192 and codon-338 truncations in the *in vitro* transcript standards are indicated. **B**) Western blot analysis of SsrA(DD) tagging. Whole-cell lysates from induced (+IPTG) and uninduced (-IPTG) cells were analyzed by Western blot using anti-SsrA(DD) polyclonal antibodies. The His_6_-GalE samples were purified by Ni^2+^-NTA affinity chromatography prior to analysis. Full-length GalE tagged at the C-terminus is indicated as **GalE-SsrA(DD)**. **C**) Mass spectrometry of SsrA(His_6_)-tagged GalE. SsrA(His_6_)-tagged GalE chains were purified as described in Methods and analyzed by electrospray ionization-mass spectrometry. SsrA(His_6_) tagging occurs at positions corresponding to GalE residues Val127 – Leu154. The identified tagging clusters are presented schematically on the GalE chain. **D**) Northern blot analysis of *galET́* and *galE*(*trp-*At) mRNA. Total RNA was isolated from tmRNA^+^ and ΔtmRNA cells and analyzed by Northern blot as described in Methods. The *in vitro* transcripts lane contains a mixture of two *galE* transcripts that are truncated at codon-192 and codon-338 of *galE*.

Roche & Sauer identified GalE as an endogenously SsrA-tagged protein in *E. coli* [21]. To study the determinants of GalE tagging, we cloned *galE* and a portion of the downstream *galT* gene into an expression vector (Fig. 1A), and overproduced GalE in cells expressing tmRNA(DD). The tmRNA(DD) variant encodes the SsrA(DD) peptide, which is resistant to proteolysis and can be readily detected by Western blot analysis [7]. Induction of GalE synthesis produced not only the previously reported full-length tagged species, but also an array of smaller SsrA(DD)-tagged chains (Fig. 1B). To determine whether these smaller products were proteolytic fragments of the full-length tagged GalE, we repeated the experiment with His_6_-GalE, which contains an N-terminal His_6_ epitope tag. Purified His_6_-GalE was tagged in essentially the same pattern as wild-type protein (Fig. 1B), indicating that GalE is tagged at a number of distinct sites. We note that GalE tagging patterns were sometimes inconsistent between blots, presumably due to variable transfer efficiencies. Therefore, all experimental samples in this work were compared to control samples run on the same blot.

To identify SsrA peptide tagging sites, we overproduced wild-type GalE in cells expressing tmRNA(His_6_). tmRNA(His_6_) encodes the SsrA(His_6_) peptide tag, which allows purification of tagged proteins by Ni^2+^-affinity chromatography [21], [23]. Purified SsrA(His_6_)-tagged GalE chains were subjected to mass spectrometry and the observed masses compared to those predicted for SsrA(His_6_)-tagged GalE chains. This analysis readily identified a number of GalE chains that were tagged between residues Val127 and Leu154 (Fig. 1C and Table S1), but failed to detect the larger SsrA(His_6_)-tagged chains observed by immunoblot. To identify these larger products, we digested SsrA(His_6_)-tagged GalE with trypsin and purified the resulting peptides by Ni^2+^-affinity chromatography for subsequent liquid chromatography-mass spectrometry. This analysis revealed dozens of tagging sites throughout the C-terminal domain of GalE (Fig. 1C and Table S1). In general, tagging occurred in distinct clusters spanning 10 to 20 residues with SsrA(His_6_) tags added after nearly every residue (Table S1).

### mRNA stability does not influence GalE tagging

All characterized examples of tmRNA•SmpB mediated tagging involve ribosomes paused on truncated mRNA. Such truncated messages are difficult to detect in tmRNA^+^ cells, but are stabilized in cells lacking tmRNA (ΔtmRNA). Presumably, in the absence of tmRNA•SmpB mediated rescue, ribosomes persist at the 3′-ends of truncated mRNA, protecting them from 3′-to-5′ exonuclease activity [5]. We examined *galET́* mRNA isolated from ΔtmRNA and tmRNA^+^ cells and detected numerous truncated transcripts, most of which were too small to account for GalE tagging (Fig. 1D). However, some truncated species were more abundant in ΔtmRNA cells compared to tmRNA^+^ cells (Fig. 1D). These species were truncated in the vicinity of codon 192 and therefore could account for the tagging cluster at residues Gly182 – Ile204 (Figs. 1C and 1D). Presumably, truncated messages corresponding to the other tagging sites are less abundant, and consequently could not be unambiguously identified by Northern blot analysis.

Aiba and colleagues have reported that translation of mRNA lacking an intrinsic transcription terminator results in SsrA tagging patterns similar to those we observe with GalE [5]. According to their model, messages lacking stable 3′-structures are susceptible to 3′-to-5′ exonucleases that degrade into the coding region and produce non-stop mRNA. Although the *galE* transcripts used here contain a 3′ stem-loop structure (the T7 transcription terminator), endonuclease cleavage within the 3′-untranslated region could remove the terminator and facilitate 3′-to-5′ exonuclease activity. In an attempt to suppress GalE tagging, we introduced an additional stem-loop structure from the *trp* operon attenuator-terminator (*trp*-At) immediately downstream of the *galE* stop codon (Fig. 1A). This approach has previously been shown to suppress SsrA tagging due to exonuclease activity [5], presumably by impeding the progress of these single-strand specific RNases. A significant proportion of *galE* transcripts contained the *trp-*At stem-loop at the 3′-terminus (Fig. 1D and data not shown), suggesting that this structure does indeed act as a barrier to exonuclease activity. However, the stem-loop had essentially no effect on the accumulation of truncated mRNA, and no effect upon GalE tagging (Figs. 1B and 1D).

**Figure 2:**
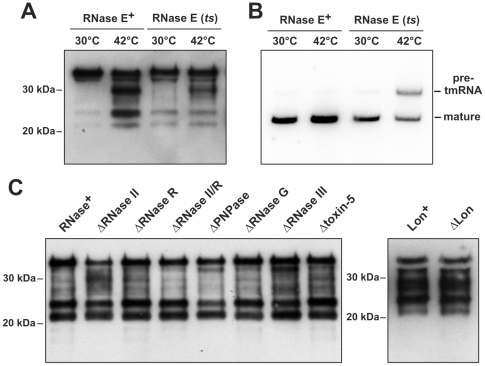
His_6_-GalE tagging in RNase deletion strains. **A**) Western blot analysis of SsrA(DD) tagging. His_6_-GalE was purified from cells expressing wild-type (RNase E^+^) or temperature sensitive RNase E(*ts*). His_6_-GalE was produced at the indicated temperatures as described in Methods. **B**) Northern blot analysis of tmRNA(DD). Total RNA was isolated from RNase E^+^ and RNase E(*ts*) cells incubated at the indicated temperatures. The migration positions of mature and pre-processed tmRNA(DD) are indicated. **C**) Western blot analysis of SsrA(DD) tagging. His_6_-GalE was purified from tmRNA(DD) cells deleted for the indicated RNases, and analyzed by Western blot using anti-SsrA(DD) polyclonal antibodies. The Δtoxin-5 strain lacks RelE, MazF, ChpBK, YafQ, and YoeB toxins. Western blot analysis was also performed on His_6_-GalE purified from tmRNA(DD) cells lacking the Lon protease.

We next sought to modulate *galE* transcript stability (and GalE tagging) by genetically manipulating the RNases that mediate mRNA turnover in *E. coli*. For these and all subsequent experiments, we used the His_6_-GalE expression construct so that equal amounts of protein purified from each genetic background could be assayed for SsrA(DD) tagging. We first examined the role of RNase E, because this endonuclease is thought to initiate the degradation of most *E. coli* messages [24], [25]. We introduced the *rne-1* allele, which encodes temperature-sensitive RNase E(*ts*) [26], into tmRNA(DD) cells and examined tagging of His_6_-GalE. His_6_-GalE tagging was somewhat reduced when RNase E(*ts*) cells were shifted to the non-permissive temperature (Fig. 2A). Although this effect could reflect stabilization of the *his_6_-galE* transcript, RNase E is also required for tmRNA maturation and activity [27]. Northern blot analysis revealed that a substantial proportion of tmRNA(DD) was not fully processed in RNase E(*ts*) cells at the non-permissive temperature (Fig. 2B). Therefore, decreased His_6_-GalE tagging could also be due to lower levels of active tmRNA(DD) in these cells. We also noted that temperature had a dramatic effect on His_6_-GalE tagging in cells containing wild-type RNase E. Although tagging of full-length His_6_-GalE was largely unaffected by temperature, tagging at all other sites was reduced in cells grown at 30°C (Fig. 2A). We also examined His_6_-GalE tagging in cells lacking two other endoribonucleases, RNase G and RNase III, which initiate the turnover of some mRNAs in *E. coli* [28], [29], [30]. However, individual deletion of either endonuclease in tmRNA(DD) cells had little effect on the tagging of His_6_-GalE (Fig. 2C).

Bacterial toxin-antitoxin (TA) systems encode small endonucleases that act as ‘mRNA interferases’ to cleave mRNA in either a ribosome-dependent or –independent manner [31], [32], [33], [34]. Because toxins are known to induce tmRNA•SmpB activity [10], [11], we examined His_6_-GalE tagging in cells deleted for five of the known *E. coli* toxin-antitoxin systems (*relBE, chpBIK, yefM-yoeB, mazEF, and dinJ-yafQ*), but observed no effect (Fig. 2C). The Gerdes and Inouye laboratories have recently identified other TA modules in *E. coli* [34], [35], so it remains possible that one or more of these RNase toxins influences GalE tagging. Because it is likely that additional TA modules will be discovered in *E. coli*, we chose to examine His_6_-GalE tagging in cells lacking the Lon protease, which is required for the activation of all known *E. coli* RNase toxins [31], [36], [37]. His_6_-GalE tagging was unaffected in ΔLon cells (Fig. 2C), suggesting that if cleaved *galE* transcripts are indeed generated by an undiscovered mRNA interferase, then the corresponding antitoxin must be degraded by another protease.

**Figure 3:**
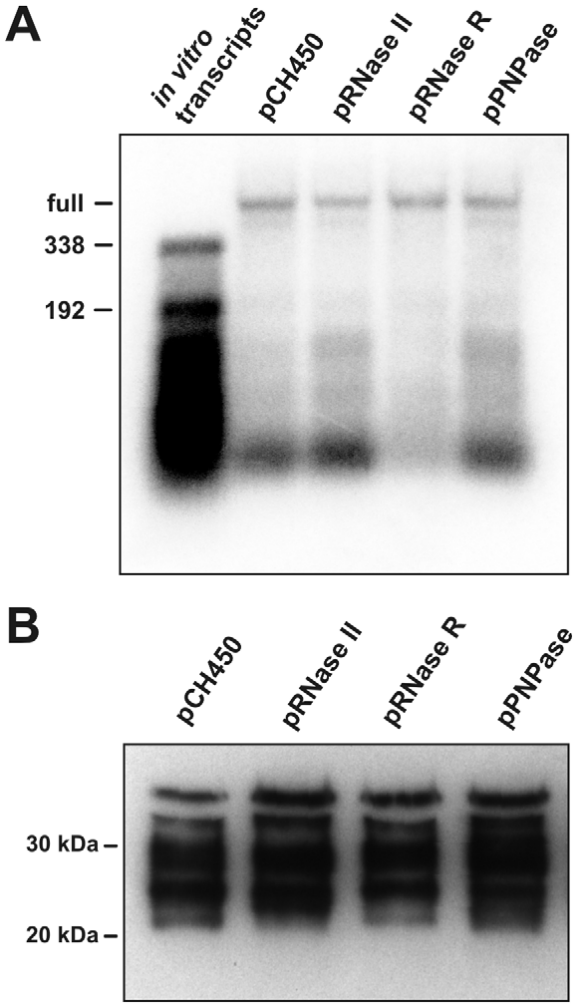
Overproduction of 3′-to-5′ exoribonucleases has no effect on His_6_-GalE tagging. **A**) Northern blot analysis of *his_6_-galE* mRNA. RNA was isolated from ΔtmRNA cells overproducing RNase II, PNPase, or RNase R from a plasmid-borne arabinose inducible promoter. Negative control RNA was isolated from cells carrying the plasmid pCH450 vector. The migration positions of *in vitro* transcripts truncated at codons-192 and -338 are indicated. **B**) Western blot analysis of SsrA(DD) tagging. His_6_-GalE was purified from tmRNA(DD) cells overproducing the indicated exonucleases, and analyzed by Western blot using anti-SsrA(DD) polyclonal antibodies. The negative control sample contains His_6_-GalE isolated from tmRNA(DD) cells carrying the plasmid pCH450 vector.

Three *E. coli* exoribonucleases – RNase II, polynucleotide phosphorylase (PNPase), and RNase R – play important roles in mRNA turnover [38]. Deletion of RNase II and RNase R individually had little effect on His_6_-GalE tagging, whereas cells lacking PNPase showed some reduction in tagging within the 27 – 34 kDa range (Fig. 2C). Tagging at these sites was also slightly reduced in cells lacking both RNase II and RNase R, but the main tagging clusters were unaffected in this background (Fig. 2C). RNase II and PNPase are reciprocally regulated, and therefore deletion of one gene results in compensatory overexpression of the remaining gene. To overcome this homeostatic regulation, we overproduced RNase II, PNPase, and RNase R from plasmid-borne inducible promoters and examined the effects on His_6_-GalE tagging. Overexpression of RNase II and PNPase increased the levels of truncated *his_6_-galE* transcripts in ΔtmRNA cells, whereas RNase R overproduction reduced the accumulation of these products (Fig. 3A). These data, as well as our previously published results [9], show that overproduced exoribonucleases influence mRNA turnover. However, there was essentially no effect on His_6_-GalE tagging when the RNases were overproduced in tmRNA(DD) cells (Fig. 3B). These results suggest that exonuclease-mediated mRNA decay plays a minor role in GalE tagging.

### GalE tagging sites do not correspond to rare codons or known translational stall sequences

**Figure 4:**
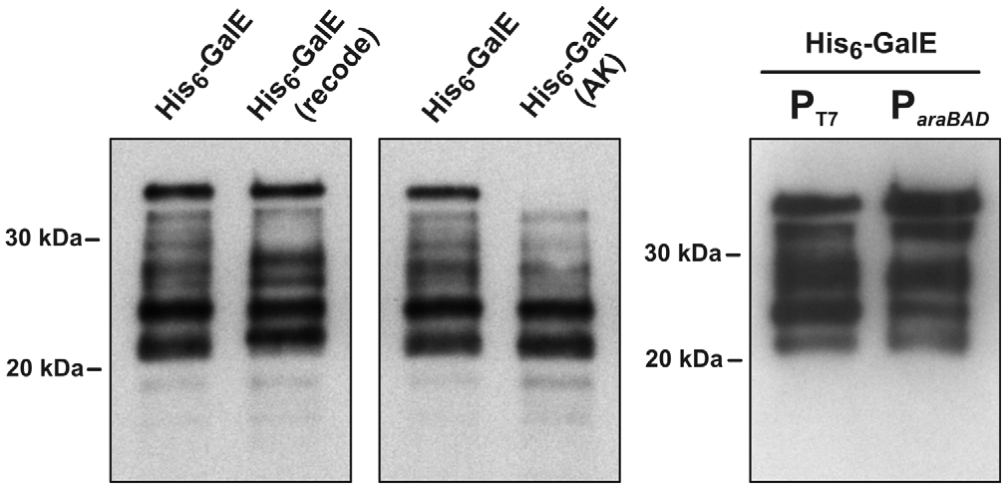
Codon usage, ribosome queuing and SsrA tagging. His_6_-GalE chains were purified from tmRNA(DD) cells and analyzed by Western blot using anti-SsrA(DD) polyclonal antibodies. The His_6_-GalE(recode) protein was expressed from a construct in which the last 170 codons had been synonymously recoded to change the mRNA sequence, but not the protein sequence (see Fig. S1). His_6_-GalE(AK) contains two additional residues (Ala-Lys) at its C-terminus. His_6_-GalE chains in the lanes labeled **P_T7_** and **P** ***_araBAD_*** were synthesized from bacteriophage T7 and *E. coli* RNA polymerase expression systems, respectively.

Several groups have demonstrated that translational pausing at rare codon clusters leads to tmRNA•SmpB activity [17], [18], [39]. The *galE* open reading frame contains five rare codons (Arg2 – AGA; Gly13 and Gly334 – GGA; Pro132 and Pro337 – CCC), but these codons are not clustered and tagging was not detected at any of these positions (Table S1). Although rare codons do not appear to play a role in GalE tagging, we tested the effects of codon usage by overproducing His_6_-GalE in cells carrying the pRARE (Novagen) plasmid. This plasmid expresses several tRNA species (tRNA_4_^Arg^, tRNA_5_^Arg^, tRNA_2_^Gly^, tRNA_2_^Ile^, tRNA_3_^Leu^, and tRNA_2_^Pro^) that are normally found at low levels in *E. coli*. Overproduction of these tRNAs had no effect on His_6_-GalE tagging (data not shown). To ensure that tRNA depletion was not responsible for translational stalling during GalE overproduction, we synonymously recoded the last 170 codons of the *his_6_-galE* ORF, corresponding to residues Phe178 – Asp338 of wild-type GalE (Fig. S1). Synonymous recoding changed some tagging in the 25–27 kDa range, but the main clusters of tagging at Gly182 – Ile204 and Gly219 – Val230 were unaffected (Fig. 4).

Full-length GalE is tagged at its C-terminus, corresponding to tmRNA•SmpB activity at ribosomes paused during translation termination. In some instances, full-length protein tagging occurs because C-terminal nascent peptide residues interfere with termination [20]. The C-terminal Pro-Asp sequence of GalE is similar to other known tagging determinants [20], and is sufficient to induce SsrA tagging of other proteins (data not shown). In principle, a ribosome paused at the *galE* stop codon could form a “roadblock” that leads to ribosome queuing. We reasoned the extensive pattern of GalE tagging could reflect tmRNA•SmpB recruitment to ribosomes paused in this manner. To test this hypothesis, we introduced two additional codons for Ala-Lys at the 3′-end of the *his_6_-galE* gene. The resulting His_6_-GalE(AK) protein was not tagged at its C-terminus (Fig. 4), suggesting that the C-terminal Ala-Lys sequence allowed efficient translation termination. However, tagging at all of the other sites was essentially identical between the His_6_-GalE and His_6_-GalE(AK) proteins (Fig. 4).

### RNA polymerase and transcription-translation coupling do not influence GalE tagging

All of the preceding experiments used bacteriophage T7 RNA polymerase (RNAP) to transcribe *galE* mRNA. T7 RNAP transcribes more rapidly than the *E. coli* polymerase, resulting in the uncoupling of transcription and translation. We hypothesized that translational uncoupling may expose the *galE* transcript to adventitious RNase activity and produce non-stop mRNA. To address this possibility, we expressed His_6_-GalE from plasmid pBAD24 using *E. coli* RNAP and observed that tagging was largely unaffected by the identity of the transcribing polymerase (Fig. 4).

### The N-terminal domain of GalE induces tagging within a heterologous C-terminal domain

**Figure 5:**
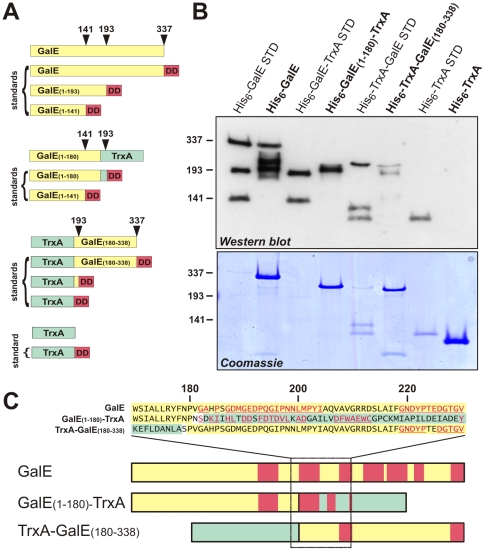
SsrA tagging of GalE-TrxA fusion proteins. **A**) Schematic representation of GalE, TrxA, and protein fusions. Also shown are SsrA(DD)-tagged standard proteins that were used as molecular markers for the gel analyses shown in panel **B**. SsrA(DD) tags were encoded at the indicated positions (downward arrows). **B**) Western blot analysis of SsrA(DD) tagging. N-terminally His_6_-tagged proteins were purified from tmRNA(DD) cells, and analyzed by Western blot using anti-SsrA(DD) polyclonal antibodies. Lanes loaded with SsrA(DD)-tagged standard proteins are indicated (**STD**). The lower panel shows a Coomassie stained polyacrylamide gel to show protein loading. **C**) Schematic representation of SsrA peptide tagging sites on GalE and fusion proteins. Amino acid residues and protein regions depicted in red indicate the sites of SsrA(His_6_) peptide tagging determined by mass spectrometry (see Supplemental data). Numbered residues correspond to the GalE primary sequence. Regions colored in yellow and light green indicate GalE and TrxA domains, respectively.

Because mRNA stability and codon usage do not influence GalE tagging, we asked whether the nascent peptide plays a role in tmRNA•SmpB activity. Nascent chain-mediated ribosome pausing has been characterized for the *E. coli* SecM and TnaC proteins [40], [41], [42], [43]. In each of these systems, relatively short nascent peptide elements interact with the ribosome exit tunnel to mediate translational arrest. Moreover, these elements are sufficient to induce ribosome arrest in other genetic contexts [42]. To test whether the GalE nascent chain interferes with translation, we generated *galE* fusion constructs with the *E. coli* thioredoxin gene (*trxA*) (Fig. 5A). TrxA was chosen for these experiments, because it increases fusion protein solubility and is not tagged by tmRNA•SmpB [20], [44]. Remarkably, in-frame fusion of the first 180 codons of *galE* to the *trxA* gene led to tagging within the C-terminal TrxA domain of the His_6_-GalE_(1–180)_-TrxA fusion protein (Fig. 5B). To identify tagging sites within the TrxA domain, we repeated the experiment in tmRNA(His_6_) cells with a fusion protein lacking the N-terminal His_6_ epitope. SsrA(His_6_)-tagged peptides were then purified and identified by mass spectrometry. This analysis revealed several tagging sites within the C-terminal TrxA domain of GalE_(1–180)_-TrxA (Fig. 5C and Table S1). Strikingly, we were unable to detect any tagging of wild-type TrxA expressed in either tmRNA(DD) or tmRNA(His_6_) cells (Fig. 5B and data not shown). In contrast, tagging within the C-terminal domain of GalE (residues 180–338) was significantly reduced when fused to an N-terminal TrxA domain (Fig. 5B). Although two of the wild-type GalE tagging clusters were observed in the TrxA-GalE_(180–338)_ fusion protein, we could not detect any other tagged products by mass spectrometry (Fig. 5C and Table S1). These data suggest that tagging at GalE residues Gly182 – Ile204, Asp238 – His257, Gly262 – Cys280, and Pro290 – Pro297 depends upon a larger genetic or molecular context.

## Discussion

The tmRNA•SmpB quality control system is recruited to stalled or otherwise inactive ribosome complexes. Ribosome stalling can occur at the 3′-ends of non-stop transcripts, or at internal sites within full-length messages. In vitro experiments indicate that tmRNA•SmpB acts only on ribosomes that are bound to truncated mRNA [22]. Therefore, translational pausing on full-length transcripts is thought to induce mRNA cleavage, which then allows tmRNA•SmpB recruitment to the paused ribosome. Truncated mRNA is invariably associated with translational pauses that induce SsrA tagging – including rare codons, inefficient stop codons, and the SecM programmed ribosome arrest [16], [23], [39], [45], [46], [47]. In each of these instances, the transcript is truncated either within the ribosomal A-site codon, or at positions that correspond to the 3′-border of the stalled ribosome. Thus, truncated mRNA can be either the cause or the consequence of ribosome arrest. Perhaps the simplest model to explain GalE tagging is one in which the translation of partially degraded *galE* transcripts causes translational arrest and subsequent tmRNA•SmpB activity. This model accounts for the findings of Yamamoto *et al.*, who reported extensive tagging of protein synthesized from mRNA lacking a stable 3′-structure [5]. In contrast, we find that GalE tagging is largely unaffected by perturbations that either stabilize or destabilize the *galE* transcript. It is particularly remarkable that overproduction of 3′-to-5′ exonucleases had no effect on GalE tagging. In principle, excess exonuclease activity could either increase tagging by adventitiously producing truncated mRNA, or decrease tagging by more rapidly clearing degradation intermediates. Taken together, these results suggest that GalE tagging is not a consequence of normal mRNA turnover. It is also somewhat surprising that deletion of RNase II had no effect on tagging, given that this 3′-to-5′ exonuclease is required for A-site mRNA cleavage during translational pauses [9]. In cells lacking RNase II, ribosome arrest produces transcripts that are truncated 12 nucleotides downstream of the A-site codon. Ribosomes stalled on such truncated transcripts are predicted to support tmRNA activity based on *in vitro* studies [22], and we find that SsrA tagging activity is unaffected in ΔRNase II cells (B.D. Janssen and C.S.H., unpublished results). These observations suggest that A-site cleavage is not strictly required for tmRNA•SmpB recruitment to paused ribosomes.

Toxin-antitoxin (TA) modules are also known to induce tmRNA•SmpB activity by virtue of their mRNA interferase activity [10], [11]. A subset of these toxins, such as RelE and YoeB, cleave mRNA in a ribosome-dependent fashion [33], [48]. Other toxins, such as MazF and ChpBK, are ribosome-independent RNases, although translated messages appear to be the preferred substrates *in vivo* [11], [49]. A prevailing model is that environmental stress activates mRNA interferases, which then cleave messages to redirect gene expression in response to the applied stress. One could imagine that toxins are activated by the stress of gratuitous GalE overproduction. However, five major *E. coli* toxins – RelE, MazF, YoeB, ChpBK, and YafQ – play no significant role in GalE tagging. Although there are additional TA modules that we have not tested, toxins are unlikely to play an important role because tagging was unaffected in ΔLon cells, which are unable to activate these systems [35]. Based on these results and our examination of other endoribonucleases, we conclude that RNase activity is not the root cause of translational pausing during GalE synthesis. This conclusion implies that ribosome pausing occurs during translation of full-length *galE* transcripts, and is followed by mRNA degradation or cleavage that then allows tmRNA•SmpB activity.

The most common mRNA determinants of ribosome pausing on full-length messages are rare codon clusters. In general, the corresponding cognate tRNAs are expressed at low levels, and therefore rare codons tend to be decoded more slowly than frequently used codons. The impact of codon usage on protein synthesis has been recognized for several decades, and it is now generally accepted that codon bias can regulate translation rates to facilitate co-translational protein folding and secretion [50], [51], [52]. Although translation of rare codons commonly elicits tmRNA•SmpB activity in *E. coli*, codon usage plays no significant role in GalE tagging. Overexpression of rare tRNA species and synonymous recoding of the *galE* gene had little to no effect on tagging. Moreover, because the primary sequence of recoded *galE* mRNA differs significantly from the wild-type sequence, these results also argue that mRNA secondary structure is not a determinant of translational pausing in this system.

Specific nascent peptide sequences also induce ribosome pausing and SsrA peptide tagging. For example, the SecM nascent peptide sequence (FxxxxWIxxxxGIRAGP) induces a site-specific translational arrest with the Pro codon positioned in the ribosomal A-site [23], [53]. Similarly, the C-terminal Pro-Pro nascent peptide motif interferes with translation termination and induces SsrA tagging in *E. coli* [20]. These nascent chain sequences act locally to pause ribosomes at specific codons; and both elements are sufficient to induce translational pausing when fused to other proteins. Tagging at the GalE C-terminus may be due to a small nascent peptide motif, because the C-terminal Pro-Asp sequence is sufficient to induce the tagging of other proteins. However, if the nascent chain induces translational pausing at the other GalE tagging sites, then the mechanism must be distinct from that of the SecM or Pro-Pro nascent chain motifs. Tagging at GalE residues Gly182 – Ile204, Asp238 – His257, Gly262 – Cys280, and Pro290 – Pro297 was not observed when the C-terminal domain was fused to TrxA. Therefore, the determinants responsible for tagging at these sites are not local sequence elements, because these sequences are not sufficient to direct tagging in other molecular contexts. Similarly, TrxA was tagged in the context of the GalE_(1–180)_-TrxA fusion protein; but wild-type TrxA was not tagged, nor was the N-terminal TrxA domain of the TrxA-GalE_(180–338)_ fusion protein. Intriguingly, tagging within the TrxA domain of GalE_(1–180)_-TrxA occurred at the same relative positions as the Gly182 – Ile204 cluster in wild-type GalE. These observations suggest that the N-terminal domain of GalE influences ribosome pausing during synthesis of the C-terminal domain.

The mechanism(s) by which the GalE N-terminal domain induces SsrA peptide tagging is still unclear. One possibility is that co-translational maturation of the N-terminus influences subsequent synthesis of the C-terminal domain. The N-terminal domain mediates GalE dimerization and also forms the NAD^+^ binding pocket [54], [55]. Perhaps problems with co-translational dimerization or the loading of NAD^+^ lead to ribosome pausing and tagging. This model provides one explanation for why the GalE N-terminus induces tagging within a fused TrxA domain, and is appealing because it provides a mechanism by which defective polypeptides could be identified and targeted for proteolysis. This model is also congruent with the observations that N-terminally fused TrxA domains increase protein expression levels in *E. coli*, and that domain order affects fusion protein expression and solubility [44], [56]. One postulate of this model is that co-translational chaperones, such as trigger factor and DnaK, could play a role in sensing the nascent protein and signaling to the ribosome. This is a particularly intriguing possibility for trigger factor, which binds to ribosomal protein L23 and is thought to interact with most nascent chains [57], [58]. However, chaperones do not appear to play a major role during GalE translation because tagging is unaffected in cells lacking trigger factor and only subtly altered cells lacking DnaK (Z.C.R. and C.S.H., unpublished results). In contrast, we find that the tagging of other large, multi-domain proteins (such as GlnRS, AcnB, and AlaRS) is dramatically increased in cells lacking the DnaK chaperone (Z.C.R. and C.S.H., unpublished results). SsrA tagging also increases in cells grown at higher temperatures, a condition that also induces protein misfolding and aggregation. Although these findings are broadly consistent with the proposed co-translational folding/maturation model, we recognize that the results are correlative and may be the result of indirect effects. Temperature affects all cellular processes, and DnaK is critical for the post-translation folding of many proteins and also has a role in ribosome assembly [59], [60]. Moreover, not all misfolding events lead to translational pausing because insoluble proteins can often be overproduced to very high levels in *E. coli*. If co-translational protein folding does indeed influence ribosome pausing, then the underlying mechanisms are likely to be complex.

## Materials and Methods

### Bacterial strains and plasmids

All strains used in this study were derivatives of *E. coli* X90 (DE3) (Table 1) [20]. Strains containing the Δ*rnc-38* and *rne-1* mutations [26], [61] were generously provided by Sydney Kushner (University of Georgia). Deletions of the *rnb, rnr, pnp, rng*, and *lon* genes were obtained from the Keio collection [62], and deletions of the *relBE, chpBIK, yefM-yoeB, mazEF,* and *dinJ-yafQ* toxin-antitoxin modules were constructed as described [45]. All gene disruptions were introduced into *E. coli* strain CH2385 [16] by bacteriophage P1-mediated transduction. The temperature-sensitive *rne-1* allele was transduced using the Keio derived Δ*yceF::kan* disruption as a linked marker. The identity of each transductant was confirmed by whole-cell PCR.

**Table 1:** Bacterial strains used in this study.

*Strain*	*Genotype^a^*	*Reference*
CH12	X90 (DE3)	[20]
CH113	X90 (DE3) *ssrA::cat*, Cm^r^	[20]
CH2182	CH12 *ssrA(DD)-kan*, Kan^r^	[16]
CH2316	CH12 *ssrA(his_6_)*	[23]
CH2385	CH12 *ssrA(DD)*	[16]
CH3136	X90 (DE3) Δ*relBE* Δ*chpBIK* Δ*yefM-yoeB* Δ*mazEF* Δ*dinJ-yafQ ssrA(DD)-kan*, Kan^r^	This study & [16]
CH3138	CH2385 *Δrnb::kan*, Kan^r^	This study & [62]
CH3139	CH2385 *Δrnr::kan*, Kan^r^	This study & [62]
CH3153	CH2385 *Δpnp::kan*, Kan^r^	This study & [62]
CH3545	CH2385 *rncΔ38::kan*, Kan^r^	This study & [61]
CH3546	CH2385 *Δrng::kan*, Kan^r^	This study & [62]
CH3547	CH2385 *ΔyceF::kan rne1*(ts), Kan^r^	This study & [26]
CH3566	CH2385 *Δrnb Δrnr::kan*, Kan^r^	This study & [62]
CH6080	CH2385 *Δlon::kan*, Kan^r^	This study & [62]

a Abbreviations used: chloramphenicol resistant, Cm^r^; kanamycin resistant, Kan^r^.

The plasmids used in this study are presented in Table 2. All T7 RNA polymerase expression constructs were derivatives of plasmid pET21b (Novagen). The *E. coli galE* and the first 47 codons of *galT* were amplified by PCR using oligonucleotides (restriction endonuclease sites are underlined), **GalE-Nde** (5′ - ATG GAG CGA CAT ATG AGA GTT CTG GTT ACC GGT GG) and **GALE REV** (5′ - CAA TCT GGA TCC TGC GCA GGT AAC ACC TGT TTG). The resulting PCR product was digested with NdeI and BamHI, and ligated to pET21b to generate plasmid pGalET́. Plasmid pGalE was generated by PCR using oligonucleotides **GalE-Nde** and **GalE-Sac** (5′ - TGG GAG CTC AAC GGG ATT AAA TTG CGT CAT GG). The *trp* attenuator stem-loop (*trp*-At) was introduced downstream of the *galE* ORF by ligation of an oligonucleotide cassette comprised of **trpAt-top** (5′ - CAG CCC GCC TAA TGA GCG GGC TTT TTT TTT TG), and **trpAt-bot** (5′ - TCG ACA AAA AAA AAA GCC CGC TCA TTA GGC GGG CTG AGC T) into SacI/SalI-digested plasmid pGalE. Plasmids pHis_6_-GalE and pHis_6_-GalE(*trp*-At) were generated by subcloning a SphI/NdeI fragment from plasmid pHis_6_-YbeL [63] into pGalE and pGalE(*trp*-At), respectively. Plasmid pHis_6_-GalE(AK) was generated by PCR amplification of pHis_6_-GalE using oligonucleotides **pET-Sph** and **galAK-rev** (5′ - TTA CTT TGC ATC GGG ATA TCC CTG TGG ATG GCG TG). The resulting PCR product was digested with SphI, and ligated to pET21b digested with BamHI, end-filled with T4 DNA polymerase, and subsequently digested with SphI.

**Table 2:** Plasmids used in this study.

*Plasmids*	*Description^a^*	*Reference*
pGalET́	pET21b-derived plasmid expressing GalE, Amp^r^	This study
pGalE(trp-At)	pET21b-derived plasmid containing the *trp* attenuator-terminator downstream of *galE*, Amp^r^	This study
pHis_6_-GalE	pET21b-derived plasmid expressing His_6_-GalE, Amp^r^	This study
pHis_6_-GalE(trp-At)	pET21b-derived plasmid containing the *trp* attenuator-terminator downstream of *his_6_-galE*, Amp^r^	This study
pHis_6_-GalE(recode)	pET21b-derived plasmid expressing His_6_-GalE from recoded *galE* gene, Amp^r^	This study
pHis_6_-GalE(AK)	pET21b-derived plasmid expressing His_6_-GalE with C-terminal Ala-Lys extension, Amp^r^	This study
pBAD-His_6_-GalE	pBAD24-derived plasmid expressing His_6_-GalE, Amp^r^	This study & [64]
pHis_6_-TrxA	pET21b-derived plasmid expressing His_6_-TrxA, Amp^r^	This study
pHis_6_-GalE_(1–180)_-TrxA	pET21b-derived plasmid expressing His_6_-GalE_(1–180)_-TrxA, Amp^r^	This study
pHis_6_-TrxA-GalE_(180–338)_	pET21b-derived plasmid expressing His_6_-TrxA-GalE_(180–338)_, Amp^r^	This study
pCH450	pACYC184-derived plasmid containing *E. coli araC* and P_araBAD_, Tet^r^	[9]
pRNase II	pCH450-derived plasmid expressing RNase II under control of P_araBAD_, Tet^r^	[9]
pPNPase	pCH450-derived plasmid expressing PNPase under control of P_araBAD_, Tet^r^	[9]
pRNase R	pCH450-derived plasmid expressing RNase R under control of P_araBAD_, Tet^r^	[9]
pRARE	pACYC184-derived plasmid expressing *E. coli* tRNA_4_^Arg^, tRNA_5_^Arg^, tRNA_2_^Gly^, tRNA_2_^Ile^, tRNA_3_^Leu^, and tRNA_2_^Pro^, Cm^r^	Novagen
pHis_6_-GalE_(1–141)_-SsrA(DD)	pET21b-derived plasmid expressing His_6_-GalE_(1–141)_-SsrA(DD), Amp^r^	This study
pHis_6_-GalE_(1–193)_-SsrA(DD)	pET21b-derived plasmid expressing His_6_-GalE_(1–193)_-SsrA(DD), Amp^r^	This study
pHis_6_-GalE_(1–337)_-SsrA(DD)	pET21b-derived plasmid expressing pHis_6_-GalE_(1–337)_-SsrA(DD), Amp^r^	This study
pHis_6_-GalE_(1–180)_-TrxA_(1–12)_-SsrA(DD)	pET21b-derived plasmid expressing His_6_-GalE_(1–180)_-TrxA_(1–12)_-SsrA(DD), Amp^r^	This study
pHis_6_-TrxA-GalE_(180–193)_-SsrA(DD)	pET21b-derived plasmid expressing His_6_-TrxA-GalE_(180–193)_-SsrA(DD), Amp^r^	This study
pHis_6_-TrxA-GalE_(180–337)_-SsrA(DD)	pET21b-derived plasmid expressing His_6_-TrxA-GalE_(180–337)_-SsrA(DD), Amp^r^	This study
pHis_6_-TrxA-SsrA(DD)	pET21b-derived plasmid expressing His_6_-TrxA-SsrA(DD), Amp^r^	This study

a Abbreviations used: ampicillin resistant, Amp^r^; chloramphenicol resistant, Cm^r^; tetracycline resistant, Tet^r^.

The last 170 codons of the *E. coli galE* gene were synonymously recoded by sequential PCR amplification of pGalET́ with the **GALE REV** reverse primer and the following forward primers: **gal-1** (5′ – AGC CGA CAC CCC CAA GGC TAC CCA GAC TAA GGA ACG ACC ATG ACG), **gal-2** (5′ – GAG ATG GCT CAA GAT ACA TGG CAT TGG CAA AGC CGA CAC CCC CAA), **gal-3** (5′ – TTA AAT TGG CGA GTC ACT CGA ACC TTA GAC GAG ATG GCT CAA GAT), **gal-4** (5′ – TGG GCT GAT GCA TCA AAG GCA GAT CGG GAG TTA AAT TGG CGA GTC), **gal-5** (5′ – CCT CGC CGA GAA GGA GAT TTA CCT GCA TAT TGG GCT GAT GCA TCA), **gal-6** (5′ – TGT GGA AAG CCT GTG AAC TAC CAC TTC GCC CCT CGC CGA GAA GGA), **gal-7** (5′ – TTA GAT GTT GTG AAC GCA TTT TCA AAG GCA TGT GGA AAG CCT GTG), **gal-8** (5′ – AAT TTA GGA GCG GGA GTC GGA AAT TCA GTT TTA GAT GTT GTG AAC), **gal-9** (5′ – TTA GCT AAT AAA CCC GGA GTC CAT ATA TAT AAT TTA GGA GCG GGA), and **gal-10** (5′ – GCT GAT GGG CAC GTC GTG GCT ATG GAG AAG TTA GCT AAT AAA CCC). The final PCR product was digested with DraIII and BamHI and ligated into pGalET́ to generate pGalE(3*′-recode*). Next, we performed sequential PCR amplification of pHis_6_-GalE with forward primer **pET-Sph** (5' - CAA GGA ATG GTG CAT GCC TGC AGA TGG CGC CC) and the following reverse primers: **recod-1** (5′ – TCC ACT AGG GTG AGC TCC CAC AGG ATT AAA GTA GCG CAG CAG GGC), **recod-2** (5′ – AGG TAT TCC CTG AGG GTC CTC TCC CAT GTC TCC ACT AGG GTG AGC), **recod-3** (5′ – CAC TTG TGC TAT ATA GGG CAT TAA ATT GTT AGG TAT TCC CTG AGG), **recod-4** (5′ – AGC TAA ACT ATC TCG CCG TCC GAC CGC CAC TTG TGC TAT ATA GGG), **recod-5** (5′ – GTC CTC TGT AGG GTA GTC ATT CCC GAA TAT AGC TAA ACT ATC TCG), **recod-6** (5′ – ATG TAT ATA GTC TCG GAC TCC CGT CCC GTC CTC TGT AGG GTA GTC), and **recod-7** (5′ – TTC CAT CGC CAC GAC GTG CCC ATC AGC TAA GTC CAT GAC ATG TAT ATA GTC TCG). The final PCR product was digested with SphI and DraIII, and ligated into pGalE(3*′-recode*) to generate plasmid pHis_6_-GalE(*recode*). Synonymous recoding was designed to change nucleotide composition as drastically as possible (see Fig. S1). Where possible, adenosine residues were changed to cytidine, and guanosine residues to uridine (and *vice versa*). For the serine and leucine six-box codons, we changed as many residues as possible. For example, serine UCG was completely changed to the synonymous AGU, and leucine UUG was recoded as CUA. However, arginine codons were not changed to either AGG or AGA, which are known to elicit ribosome pausing and tmRNA tagging activity.

The *galE_(1-180)_* fragment was generated by PCR using oligonucleotides **pET-Sph** and **M1-P180-Eco** (5′ - CGC GGA ATT CGG GTT GAA GTA GCG CAG CAG). *E. coli trxA* was amplified using oligonucleotides **trxA-Eco(for)** (5′ - ATA GAA TTC CGA TAA AAT TAT TCA CCT GAC) and **trxA-BamHI** (5′ - GAG GAT CCC TTA CGC CAG GTT AGC GTC GAG), and ligated downstream of the *galE_(1–180)_* fragment in plasmid pET21b to generate plasmid pGalE_(1–180)_-TrxA. The *trxA-galE_(180–338)_* fusion was generated by PCR of *trxA* with **trxA-Nde** (5′ - GTG GAG TTA CAT ATG AGC GAT AAA ATT ATT CAC C) and **trxA-Nhe-rev** (5′ - TTA GCT AGC CAG GTT AGC GTC GAG G); and amplification of *galE_(180–338)_* with **galE-P180-Nhe** (5′ - ATT GCT AGC CCG GTT GGC GCG CAT CCG) and **GALE REV**. The resulting PCR products were digested with the indicate restriction endonucleases and sequentially ligated into pET21b. These fusions were subcloned into plasmid pHis_6_ following digestion by NdeI and BamHI. pHis_6_-TrxA was generated by PCR of *trxA* with oligoribonucleotides **trx-Nde** and **trxA-BamHI**, followed by digestion with NdeI and BamHI, and ligation into pHis_6_. The NcoI/SalI fragment from pHis_6_-GalE was subcloned into pBAD24 [64], to generate plasmid pBAD-His_6_-GalE. The sequences of all plasmid constructs were confirmed by DNA sequencing.

### Analysis of tmRNA-mediated peptide tagging

Overnight *E. coli* cultures were resuspended to OD_600_ = 0.05 in fresh LB medium containing the appropriate antibiotics (150 µg/ml ampicillin; 12.5 µg/ml tetracycline; 50 µg/ml kanamycin; 66 µg/ml chloramphenicol) and incubated at 37°C with aeration. Once cultures reached OD_600_ ∼0.4–0.5, GalE (and its derivatives) production was induced with 2 mM isopropyl β-D-thiogalactopyranoside (IPTG) for pET-derived plasmids, or with 0.4% L-arabinose for pBAD24-derived plasmids. Cells were harvested by centrifugation 90 min after induction and cell pellets frozen at −80°C. Urea-soluble lysates were prepared by extracting frozen cells with 8 M urea – 150 mM NaCl – 5 mM β-mercaptoethanol – 0.5% Triton X-100 – 10 mM Tris-HCl (pH 8.0) as described previously [17]. Proteins containing N-terminal His_6_ or C-terminal SsrA(His_6_) tags were purified by Ni^2+^-nitrilotriacetic acid (Ni^2+^-NTA) affinity chromatography as described [17]. Urea-soluble cell lysates and Ni^2+^-NTA purified peptides were analyzed by SDS-PAGE and Western blot using Tris-tricine buffered 10% polyacrylamide gels and semi-dry electrotransfer to Immobilon PVDF membranes (Millipore). Immunoblotting using rabbit anti-SsrA(DD) antibodies was performed as described [20].

Ni^2+^-NTA purified proteins were prepared for mass spectrometry by two methods. First, purified His_6_-tagged proteins were fractionated by HPLC as described [17]. Fractions were dried by speed-vac and reconstituted in aqueous 50% acetonitrile – 1% formic acid and injected directly into a Waters Q-Tof II™ mass spectrometer. Mass data were processed using MassLynx analytical software. Second, purified SsrA(His_6_)-tagged proteins were fractionated and dried as described above. Fractions were reconstituted in 2 M urea – 1 mM CaCl_2_ – 100 mM Tris-HCl (pH 8.45), followed by digested with 1 mg/ml trypsin for 16 hours at 37°C. Tryptic digests were quenched by addition of 5 volumes of 8 M urea – 150 mM NaCl – 5 mM β-mercaptoethanol – 0.5% Triton X-100 – 10 mM Tris-HCl (pH 8.0). SsrA(His_6_)-containing tryptic fragments were re-purified by Ni^2+^-affinity chromatography and then applied to a Zorbax 300SB-C18 reverse phase column in aqueous 0.1% formic acid and proteins eluted using a linear gradient of acetonitrile using an Agilent 1100 LC nano-system. Eluted proteins were infused into a Waters Q-Tof II™ mass spectrometer for mass determination.

### RNA analyses

For RNA isolation, cultures were poured into an equal volume of ice-cold methanol 90 min after induction. Cells were then collected by centrifugation and cell pellets frozen at −80°C. Total RNA was isolated from frozen cell pellets using acidic guanidine isothiocyanate/phenol as described previously [23]. Northern blot analysis of tmRNA was performed using 50% urea – 6% polyacrylamide gels and electrotransfer to Nytran SPC nylon membranes as described [45]. Oligonucleotide **SsrA probe** (5′ - TGG TGG AGC TGG CGG GAG TTG AAC) was radiolabeled and used a hybridization probe. Northern blot analysis of *galE* transcripts was conducted using glyoxal denatured RNA and 1% agarose gels as described [65]. RNA was transferred to nylon membranes by capillary transfer and probed with radiolabeled **T7-SD probe** (5′ - GTA TAT CTC CTT CTT AAA GTT AAA C). *In vitro* transcription using bacteriophage T7 RNA polymerase was performed as described previously [45]. The *galE-192* transcription template was generated by PCR using oligonucleotides **pET-Sph** and **galE-trunc** (5′ - ATT CGG AAT GCC TTG CGG ATC TTC). The *galE-338* transcription template was generated using oligonucleotides **pET-Sph** and **galE-stop-trunc** (5′ - TAA TCG GGA TAT CCC TGT GGA TGG CG).

## Supporting Information

Figure S1 **Alignment of wild-type and recoded** ***galE*** **open reading frames.** The last 170 codons of the ***E. coli*** ** ***galE*** gene were synonymously recoded as described in the Materials and Methods. Mutated residues are indicated by red blocks. The encoded polypeptide is presented in one-letter amino acid code.
(TIF)

Table S1 **Mass spectrometry analysis of tmRNA-tagged peptides.** Peptides were purified and analyzed as described in Materials and Methods. Predicted masses were computed using the Compute pI/MW online tool (http://expasy.org/tools/pi_tool.html). Sequences used for the predictions were generated by creating *in silico* fusions of truncated GalE tryptic peptides to the C-terminal SsrA(His_6_) peptide tag (AANDHHHHHHD).
(XLS)
