# Supplementary figures and images for: The N-Terminus of GalE Induces tmRNA Activity in Escherichia coli
Source: PLoS One. 2010 Dec 7;5(12):e15207. doi: 10.1371/journal.pone.0015207 (PMC2998420; doi:10.1371/journal.pone.0015207)

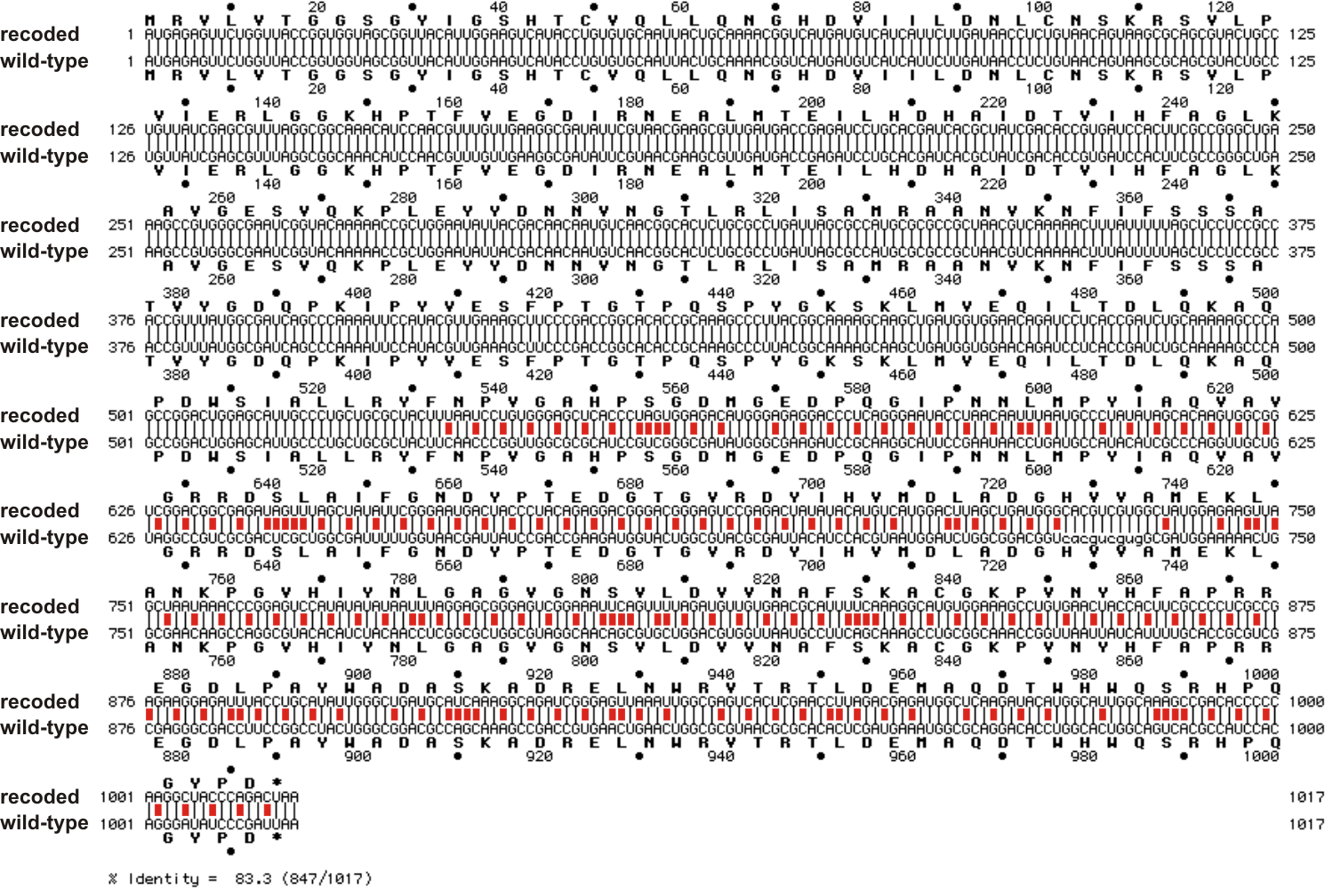

Supplement: Figure S1 — Alignment of wild-type and recoded galE open reading frames. The last 170 codons of the E. coli galE gene were synonymously recoded as described in the Materials and Methods. Mutated residues are indicated by red blocks. The encoded polypeptide is presented in one-letter amino acid code. (TIF) [file pone.0015207.s001.tif]
